# Berbamine inhibits SARS-CoV-2 infection by compromising TRPMLs-mediated endolysosomal trafficking of ACE2

**DOI:** 10.1038/s41392-021-00584-6

**Published:** 2021-04-24

**Authors:** Lihong Huang, Terrence Tsz-Tai Yuen, Zuodong Ye, Shuyan Liu, Guoliang Zhang, Hin Chu, Jianbo Yue

**Affiliations:** 1 City University of Hong Kong Shenzhen Research Institute, Shenzhen, China; 2 Department of Biomedical Sciences, City University of Hong Kong, Hong Kong, China; 3 Department of Microbiology, Li Ka Shing Faculty of Medicine, The University of Hong Kong, Hong Kong, China; 4 National Clinical Research Center for Infectious Diseases, Shenzhen Third People’s Hospital, Shenzhen, China

**Keywords:** Microbiology, Cell biology, Target identification

**Dear Editor**,

Middle East respiratory syndrome-related coronavirus (MERS-CoV) is the pathogen responsible for the outbreak of MERS, and we are currently being affected by coronavirus disease 2019 (COVID-19) due to infection by the severe acute respiratory syndrome coronavirus 2 (SARS-CoV-2). The S protein of SARS-CoV-2 or MERS-CoV binds angiotensin-converting enzyme 2 (ACE2) or dipeptidyl peptidase-4 (DPP4), respectively, to facilitate viral particles entry into cells^1^. The COVID-19 pandemic has caused major socioeconomic disruptions globally.

The Ca^2+^ signaling has been reported to be essential for virus entry^2^, and berbamine, a bis-benzylisoquinoline alkaloid, modulates Ca^2+^ signaling both in intro and in vivo^3^. We showed that berbamine effectively inhibited the entry of SARS-CoV-2-S or MERS-CoV-S pseudotyped particles into host cells (Fig. 1a, Supplememtary Fig. S1a–S1c). We subsequently found that berbamine significantly decreased both the intracellular (Supplememtary Fig. S1d) and extracellular (Supplememtary Fig. S1e) levels of MERS-CoV RNA in primary human lung fibroblasts. We also assessed the anti-SARS-CoV-2 activity of berbamine in Vero-E6 cells, and found that berbamine significantly inhibited viral yield, as quantified by qRT-PCR assays (EC_50_ = ~2.4 μM) (Fig. 1b) or virus titration assays (Supplememtary Fig. S1f). In summary, these data indicate that berbamine is a potential drug against SARS-CoV-2 and MERS-CoV.

**Fig. 1:**
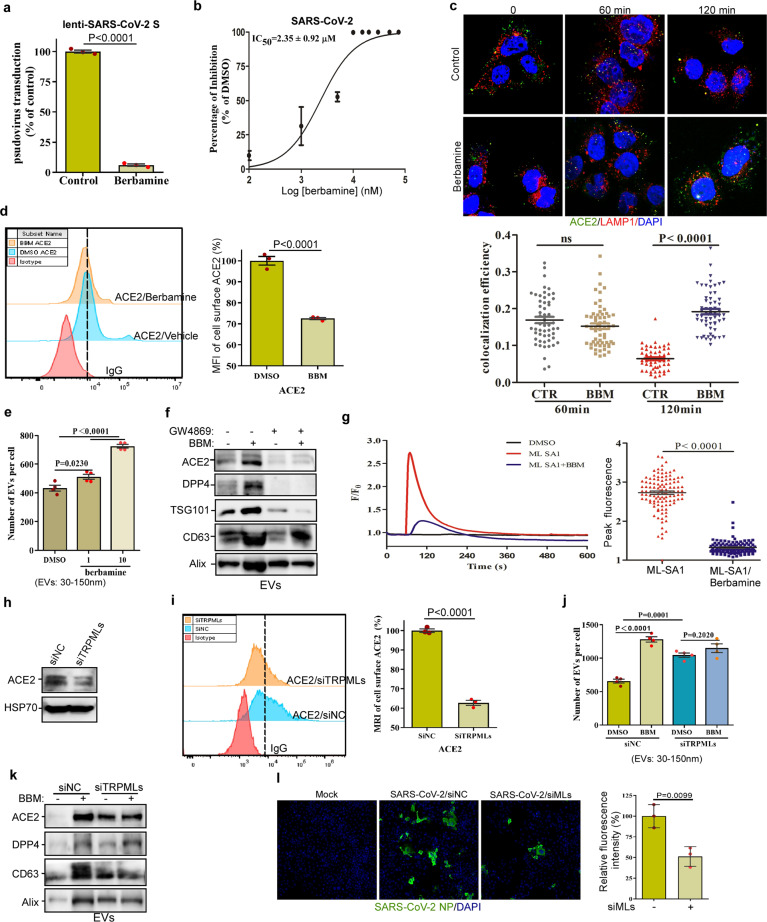
Berbamine inhibits SARS-CoV-2 infection by compromising TRPMLs-mediated endolysosomal trafficking of ACE2. **a** Berbamine (10 μM) inhibited the entry of lenti-SARS-CoV-2 S pseudotyped particles into hACE2-overexpressed HEK293T cells. **b** Vero-E6 cells were treated with berbamine at the indicated concentrations for 3 h, and then they were then infected with SARS-CoV-2 (~0.01 MOI). The cell lysates were collected and subjected to RT-PCR quantification of SARS-CoV-2 RNA. **c** Huh7 cells, treated with or without berbamine (10 μM), were first incubated with an ACE2 antibody on ice for 90 min, and the internalization of the ACE2-antibody complex was then initiated at 37 °C for the indicated times, followed by LAMP1, and DAPI staining and confocal imaging. The colocalization efficiency between ACE2 and LAMP1 was quantified. **d** Huh7 cells were treated with or without berbamine (10 μM) for 24 h, and the live cells were immunolabeled with the anti-ACE2 antibody, followed by FACS analysis to measure the cell surface ACE2 levels. **e**, **f** EVs were collected from the culture medium of control or berbamine (10 μM)-treated Huh7 cells in the presence or absence of GW4689, and their concentration and distribution of sizes were determined with a nanoparticle tracking analyzer (**e**). The levels of ACE2, DPP4, TSG101, and CD63 in these EVs were determined by immunoblot analysis (**f**). **g** Berbamine (BBM) significantly inhibited the ML-SA1-induced cytosolic Ca^2+^ increase in GECO-TRPML1-expressing HEK293T cells. **h** Cells were transfected with siRNA pools against all TRPMLs for 48 h, and the cell lysates were then subjected to ACE2 immunoblot analysis. **i** TRPMLs knockdown significantly inhibited the cell surface ACE2 levels in Huh7 cells as determined by FACS analysis. **j**, **k** EVs were collected from the culture medium of control or TRPMLs-knockdown Huh7 cells, and their concentration and distribution of sizes were determined with a nanoparticle tracking analyzer (**j**). The levels of ACE2, DPP4, and CD63 in these EVs were determined by immunoblot analysis (**k**). **l** TRPMLs knockdown inhibited SARS-CoV-2 infection in Huh7 cells as shown by SARS-CoV-2 nucleocapsid protein (NP) immunostaining. The graphs represent data from at least three independent experiments. The difference between two groups was analyzed using two-tailed Student’s *t*-test, *P* < 0.05 was considered statistically significant

Interestingly, berbamine significantly inhibited the ability of Gly-Phe β-naphthylamide (GPN) to trigger Ca^2+^ release from lysosomes (Fig. S2a), which suggests that it inhibits lysosomal Ca^2+^ channels. Since lysosomal Ca^2+^ channels participate in various endolysosomal trafficking events^4^, it is possible that berbamine might inhibit these channels to compromise the trafficking of ACE2, thereby preventing the entry of the virus. We, thus, examined whether berbamine changes the trafficking of ACE2. Briefly, cells were first incubated with an ACE2 antibody on ice for 90 min, and the internalization of the ACE2-antibody complex was then initiated at 37 °C. In control cells, within 60 min, the ACE2-antibody complex had re-localized from the cell membrane to the late endosomes or lysosomes, as manifested by the co-localization of ACE2 and LAMP1, a late endosome/lysosome marker. After ~2 h, the majority of the internalized ACE2-antibody complex was degraded in control cells (top panel in Fig. 1c). In contrast, the endolysosomal trafficking of the ACE2-antibody complex in berbamine-treated cells was significantly delayed when compared to the control cells (bottom panel in Fig. 1c), suggesting that berbamine inhibits the endosomal trafficking of ACE2. We reasoned that the inhibition of ACE2 endosomal trafficking by berbamine might affect its levels at the cell surface. By immunolabeling ACE2 in cells treated with or without berbamine followed by flow cytometric analysis, we showed that berbamine significantly decreased the levels of ACE2 at the plasma membrane (Fig. 1d). Similarly, berbamine treatment significantly decreased the levels of DPP4 at the plasma membrane (Supplememtary Fig. S2b). These results suggest that berbamine prevents SARS-CoV-2 or MERS-CoV from entering host cells by decreasing the levels of ACE2 or DPP4 at the plasma membrane. In addition, we showed that berbamine had little effect on the integrity of the cell plasma membrane (Supplememtary Figs S2c and S2d) and exhibited low cytotoxicity (Supplememtary Fig. S2e).

Interfering endolysosomal trafficking has been shown to promote the exosome release^5^. As expected, berbamine significantly promoted the secretion of extracellular vesicles (EVs) in Huh7 cells as quantified by a nanoparticle analyzer (Fig. 1e). We then examined whether these EVs contain elevated levels of ACE2 or DPP4 in the berbamine-treated group when compared with the control group. Indeed, the levels of ACE2 and DPP4, similar to other exosome protein markers, e.g., TSG101, CD63, and Alix, were markedly increased in EVs collected from the berbamine-treated cell culture medium when compared with the control group (Fig. 1f and S2f). Whereas GW4869, a sphingomyelinase inhibitor that can abolish the secretion of exosome not the EVs budding from plasma membrane, abolished the levels of ACE2 and DPP4 in EVs induced by berbamine (Fig. 1f). These results indicate that berbamine induces the secretion of DPP4 and ACE2 via exosomes. We reasoned that the increase in the secretion of ACE2 and DPP4-containing exosomes from cells might lead to the reduced levels of these receptors in berbamine-treated cells. Indeed, when compared with the control cells, berbamine treatment of cells markedly decreased the levels of ACE2 and DPP4 (Supplememtary Fig. S2g). These results suggest that berbamine inhibits the endolysosomal trafficking of ACE2. This leads to an increase in ACE2 secretion via exosomes and a concomitant decrease in its level at the plasma membrane.

Since transient receptor potential mucolipin channels (TRPMLs) are one class of main Ca^2+^-permeable channels in lysosomes, we assessed whether berbamine modulates TRPMLs-mediated Ca^2+^ release from lysosomes. We transfected HEK293T cells with GECO-TRPML1, a lysosome-targeted Ca^2+^ sensor, and treated cells with ML-SA1, a selective and potent TRPMLs agonist. ML-SA1 markedly induced the lysosomal Ca^2+^ release, and this ML-SA1-induced Ca^2+^ increase was significantly inhibited by berbamine treatment (Fig. 1g). In TRPML1^L15L/AA-L577L/AA^-expressing HEK293 cells, TRPML1-GFP was retouted to the plasma membrane. ML-SA1 markedly induced Ca^2+^ influx, whereas berbamine significantly inhibited this Ca^2+^ influx (Supplememtary Fig. S2h). These results indicated that berbamine is a potential TRPMLs inhibitor. We then knocked down the expression of TRPML1, 2, and 3 simultaneously by pools of siRNAs against TRPMLs in Huh7 cells (Supplememtary Fig. S2i), and showed that TRPMLs knockdown, similar to berbamine treatment (Supplememtary Fig. S2g), markedly decreased the levels of ACE2 in Huh7 cells (Fig. 1h). Also, TRPMLs knockdown significantly decreased the levels of ACE2 and DPP4 at the cell surface (Fig. 1i and Supplememtary Fig. S2j). Consistently, TRPMLs knockdown significantly increased EVs secretion in Huh7 cells (Fig. 1j), and markedly increased the levels of ACE2, DPP4, CD63, and ALIX in exosomes collected from the knockdown cells when compared to the control cells (Fig. 1k). Notably, in TRPMLs-knockdown cells, berbamine treatment failed to further increase EV secretion or ACE2 expression in EVs (Fig. 1j and k). Finally, we assessed the role of TRPMLs in SARS-CoV-2 infection. We infected the control or TRPMLs-knockdown Huh7 cells with SARS-CoV-2, followed by SARS-CoV-2 nucleocapsid protein (NP) immunostaining. We showed that TRPMLs significantly inhibited SARS-CoV-2 infection in Huh7 cells, manifested by fewer SARS-CoV-2 NP-positive TRPMLs-knockdown cells when compared to the control cells (Fig. 1l). In summary, these data indicate that berbamine compromises the endolysosomal trafficking of ACE2 via inhibition of TRPMLs, and this leads to an increase in the secretion of ACE2 via exosomes and a concomitant decrease in the levels of ACE2 at the cell surface, thereby preventing SARS-CoV-2 from entering the host cells. Therefore, berbamine, a prescribed drug for treating leukopenia in cancer patients in China for years, is a potential and attractive therapeutic agent for the prevention and/or treatment of SARS-CoV-2 infection.

## Supplementary information

Materials and methods, supplementary figures and tables

## Data Availability

All supporting data are included in the manuscript and Supplemental files. Additional data are available upon reasonable request to the corresponding author.
