# Supplementary material for: Berbamine inhibits SARS-CoV-2 infection by compromising TRPMLs-mediated endolysosomal trafficking of ACE2
Source: Signal Transduct Target Ther. 2021 Apr 24;6:168. doi: 10.1038/s41392-021-00584-6 (PMC8065329; doi:10.1038/s41392-021-00584-6)
Supplement: Supplementary file 1 — Materials and methods, supplementary figures and tables [file 41392_2021_584_MOESM1_ESM.docx]

Supplementary Materials for

**Berbamine inhibits SARS-CoV-2 infection by compromising TRPMLs-mediated endolysosomal trafficking of ACE2**

Lihong Huang^1, 2^*, Terrence Tsz-Tai Yuen ^3^*, Zuodong Ye^1, 2^*, Shuyan Liu^4^*, Guoliang Zhang^4^, Hin Chu^3#^, Jianbo Yue^1, 2#^

Correspondence to: [hinchu@hku.hk](mailto:hinchu@hku.hk) & [yue.jianbo@cityu.edu.hk](mailto:yue.jianbo@cityu.edu.hk)

**This PDF file includes:**

Materials and Methods

Figures. S1 to S2

Tables S1 to S2

Materials and Methods

***Cell culture and virus propagation-*** VeroE6, Huh7, and HEK293T cells were maintained in DMEM (Gibco, 12800082) containing 10% fetal bovine serum (Gibco, 10500064) and 100 U/ml of penicillin/streptomycin. SARS-CoV-2 (strain HKU001, GenBank accession no MT230904) was obtained from the nasopharyngeal aspirate of a COVID-19 patient from Hong Kong as we previously described ^30^. MERS-CoV was a gift from Dr. Ron Fouchier (Erasmus Medical Center, Rotterdam, the Netherlands). SARS-CoV-2 and MERS-CoV were expanded in Vero-E6 cells. Virus titers were determined by plaque assays as we previously described ^31^. All experiment protocol follows the standard operating procedures of Biosafety Level 3 facility at the Department of Microbiology, University of Hong Kong.

***Plasmids-*** psPAX2 (Addgene # 12260) and pMD2.G (Addgene # 12259) were ordered from Addgene. The GECO-TRPML1 was a gift from Dr. Dong Xianping at Dalhousie University.

*Production of pseudotyped coronavirus particle-* HEK293T cells were co-transfected with a SARS-CoV-2 S or MERS-CoV S encoding-plasmid, a lentivirus Gag-Pol packaging plasmid, and a lentivirus transfer vector encoding RFP-tagged Histone B gene or luciferase reporter gene by using linear polyethyleneimine (PEI). pcDNA3.1-SARS-CoV2-Spike was ordered from Addgene (Addgene# 145032) and pTG-Luc pCAGGS-MERS-S was generously provided by Prof. Gary R. Whittaker. Cells were incubated with transfection medium for 4 h at 37℃. After washing, cells were incubated with DMEM containing 10% FBS for another 60 h. The supernatants were then harvested, filtered through 0.45-mm membranes, concentrated with a 30 kDa membrane for 30 min at 5,000 rpm, and stored at -80℃.

***The anti-SARS-CoV-2 activity of berbamine-*** Vero-E6 cells were pre-treated with berbamine at a titration of different concentrations (0-75 μM) for 6 hours. Then, the cells were washed with PBS and inoculated with SARS-CoV-2 at 0.01 MOI for 2 hours. At 2 hours post-infection (h.p.i.), the cells were washed with PBS and treated with berbamine at a titration of different concentrations (0-75 μM). At 48 h.p.i., 100 μL of viral supernatant was lysed and proceed to total RNA extraction using the QIAamp viral RNA mini kit (Qiagen, Hilden, Germany). The extracted RNA was then used to quantify the replication of SARS-CoV-2 using real-time quantitative RT-PCR (qRT-PCR) as described previously ^30^. Alternatively, virus-containing supernatants with serial 10-fold dilution were used to infect Vero-E6 cells and the Tissue Culture Infectious Dose 50% (TCID_50_) was determined in a microtitration assay by the Reed-Muench method.

***Western blot analysis****-* The Bradford assay (Bio-RAD) was performed to measure the protein concentration of cell lysates. An equal amount of protein sample was loaded onto 8%-12% SDS-PAGE gels for electrophoresis. The proteins were then transferred to a PVDF membrane (Millipore), blocked with 5% non-fat milk, and blotted with primary and secondary antibodies. The primary antibodies used for immunoblotting are listed in **Table S1**.

***Cell surface receptors staining for flow cytometry*-** Cells were harvested by trypsinization and were resuspend cells in PBS. Cells were then incubated with primary antibodies on ice for 90 min, followed by incubation with the secondary antibody at room temperature for 30 min. After washing, the samples were subjected to flow cytometry analysis to quantify the levels of the receptors at the cell surface.

***Purification of extracellular vesicles from the culture medium-*** Huh7 cells were grown in 15-cm dishes to ~80% confluency. The cells were then rinsed with PBS and incubated in an EV-depleted complete medium containing DMSO or berbamine (25 μM) for 48 h. The supernatant was then collected and subjected to sequential centrifugation steps at different centrifugal forces (g) to remove the intact cells, dead cells, or cell debris. After each centrifugation, the supernatant was transferred into a new 50 mL tube and the pellet was discarded. Finally, the supernatant was subjected to ultracentrifugation at 120,000 × g for 90 min, and the pellet (containing EVs) was washed with PBS and subjected to another ultracentrifugation at 120,000 × g for 90 min. Finally, the EVs pellets were collected and subjected to immunoblot analysis.

***Intracellular Ca^2+^ measurements-*** Huh7 cells were grown in 24-well plates to ~80% confluency. The cells were then loaded with HBSS (Gibco, 14025092) containing 4 μM Fura-2 AM (Invitrogen, F1221) and 0.4% Pluronic™ F-127 (Invitrogen, P3000MP) at room temperature for 30 min. The cells were washed with Ca^2+^-free HBSS containing 2 mM EGTA and incubated in Ca^2+^-free HBSS in the presence or absence of berbamine (25 μM) at room temperature for another 30 min. Fluorescence images were acquired at 3 s intervals by alternate excitation at 340 nm and 380 nm with emission at 510 nm using a Nikon Eclipse Ti-S Calcium imaging system. Approximately 1 min after live-cell imaging, 250 μM GPN (Abcam, ab145914) was added to the cells to trigger Ca^2+^ release from the lysosomes.

***GECO Ca^2+^ imaging*-** HEK293T cells were transiently transfected with GECO-TRPML1. 24 h after transfection, the cells were washed with Ca^2+^-free HBSS containing 2 mM EGTA and incubated in Ca^2+^-free HBSS in the presence or absence of berbamine (25 μM) at room temperature for another 30 min. Fluorescence images were acquired at 3 s intervals by alternate excitation at 470 nm using a Nikon Eclipse Ti-S Calcium imaging system. Approximately 1 min after live cell imaging, 25 μM ML-SA1 (Tocris Bioscience, 4746) was added to the cells to trigger Ca^2+^ release from the lysosomes.

***ML-SA1-induced* *Ca^2+^ influx in TRPML1^L15L/AA-L577L/AA^-expressing HEK293T cells****-* HEK293T cells were transfected with GFP tagged TRPML1-L15L/AA-L577L/AA (ML1-4A) for 24 h, followed by the treatment with berbamine (25 μM) for 3 h. The cells were then loaded with HBSS containing 4 μM Fura-2-AM at room temperature for 30 min. Afterward, the cells were incubated in Ca^2+^-containing HBSS or Ca^2+^-free HBSS in the presence or absence of berbamine (25 μM) at room temperature for another 30 min. Fluorescence images were acquired at 3 s intervals by alternate excitation at 340 nm and 380 nm with emission at 510 nm using a Nikon Eclipse Ti-S Calcium imaging system. Approximately 1 min after live-cell imaging, ML-SA1 (10 μM) was added to the cells to trigger Ca^2+^ influx.

***Small interference RNA (siRNA)-*** Cells were transfected with siRNAs against respective genes (**Table S2**) using Lipofectamine RNAimax according to the manufacturer’s instructions. The knockdown efficiency was validated by qRT-PCR analysis.

***Immunofluorescence staining-*** Cells were fixed with 4% paraformaldehyde (PFA) solution, blocked with PBS containing 5% normal donkey serum and 0.3% Triton™ X-100, and then incubated with primary antibody followed by the appropriate fluorescent secondary antibody. To label the receptors on the plasma membrane, live cells were incubated with the primary antibody in PBS (+1% BSA) on ice for 90 min, followed by incubation with the fluorescent secondary antibody on ice. Images were captured with a Carl Zeiss LSM 880 confocal microscope using a 63×oil objective lens. The primary antibodies used in these experiments are listed in **Table S1**.

***Statistical analysis****-* Data are presented as mean ± S.E.M. Statistically significant differences were determined by the Student’s t-test and *P* < 0.05 was considered to be statistically significant.

Figure. S1.


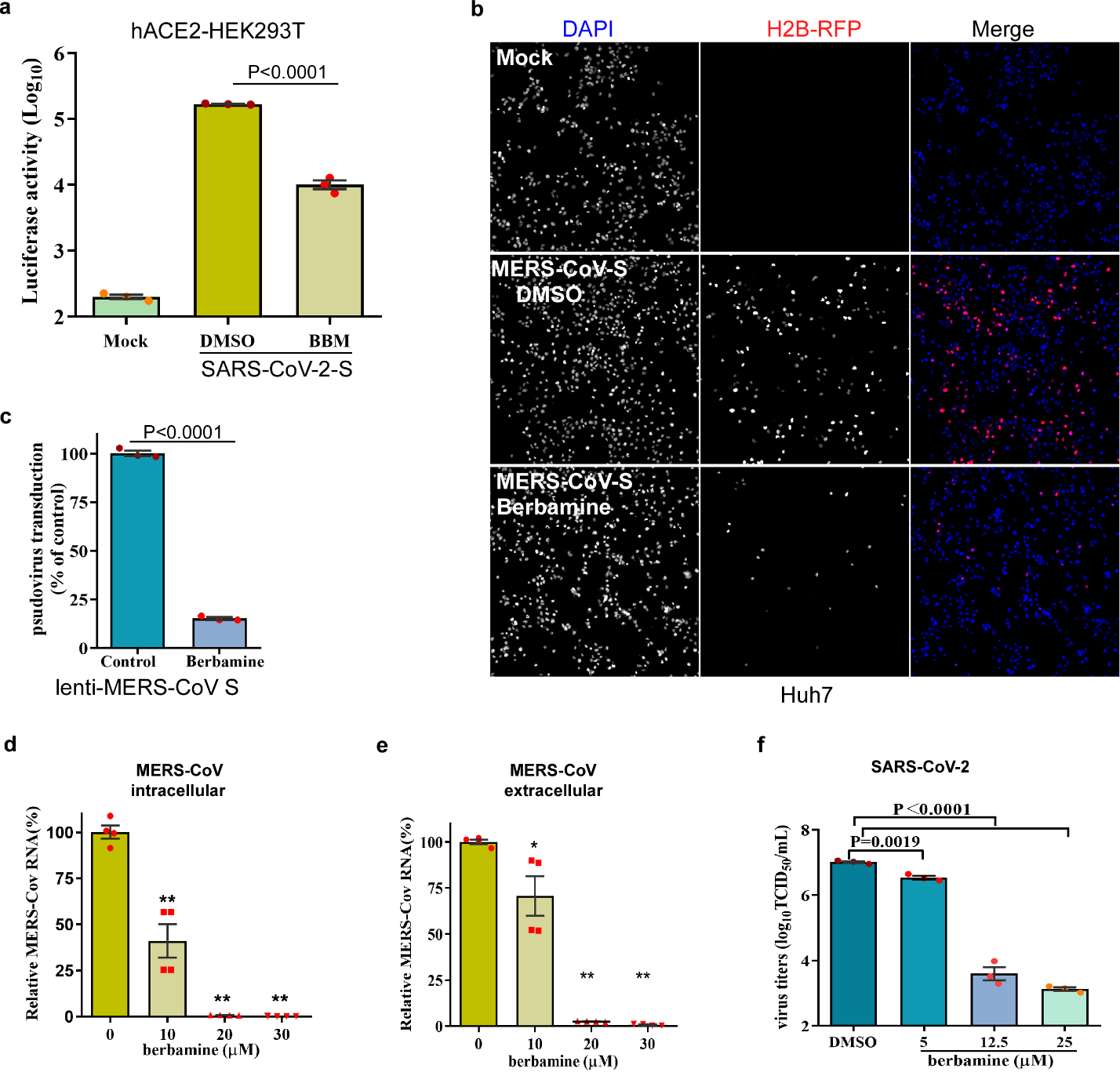
 **Figure S1. Berbamine inhibits the entry of SARS-CoV-2-S or MERS-CoV-S infection of host cells.** (**A-C**) Berbamine (10 μM) inhibited the entry of Lenti-SARS-CoV-2-S (**A**) or Lenti-MERS-CoV-S (**B, C**) pseudotyped particles in hACE2-overexpressed HEK293T or Huh7 cells, respectively. The images and graphs represent data from three independent experiments. **(D**, **E)** Primary human lung fibroblast cells were treated with berbamine at the indicated concentration for 3 h, after which they were infected with MERS-CoV (~0.1 MOI). Cell lysates (**D**) and supernatant (**E**) were collected separately and subjected to RT-PCR quantification of MERS-CoV RNA. (**F**) Vero-E6 cells were treated with berbamine at the indicated concentrations for 3 h, and then they were then infected with SARS-CoV-2 (~0.01 MOI). The cell lysates were collected and subjected to virus titer measurement. The graphs represent data from at least three independent experiments. The difference between two groups was analyzed using two-tailed Student's t-test, P<0.05 was considered statistically significant.

Figure. S2.


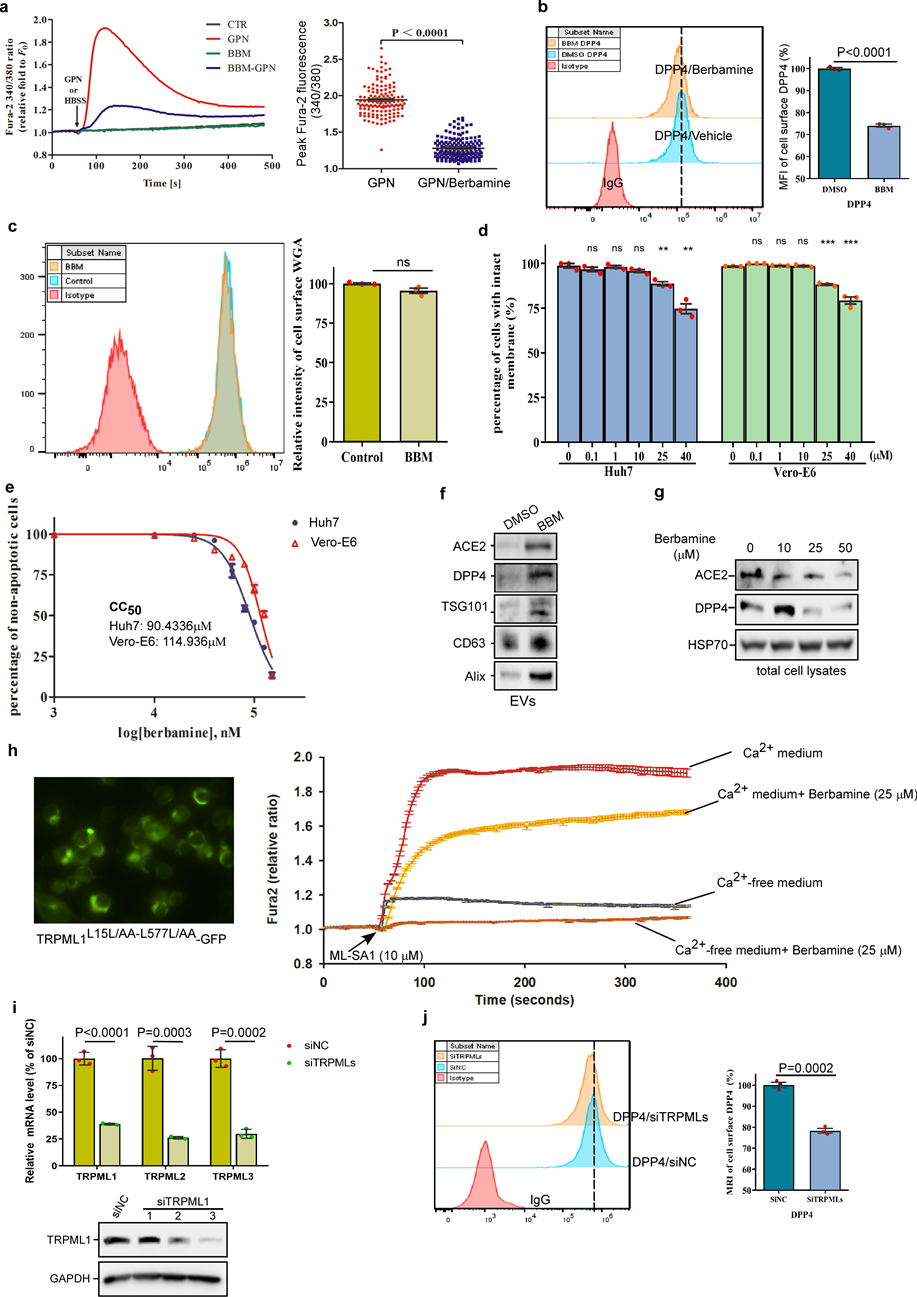


**Figure S2. Berbamine inhibits Ca^2+^ release from the lysosome to compromise endolysosomal trafficking of ACE2 and induce its secretion in EVs. (A**) Berbamine (BBM) significantly inhibited the GPN-induced cytosolic Ca^2+^ increase in Fura-2-loaded Huh7 cells. (**B**) Huh7 cells were treated with or without berbamine (10 μM) for 24 h, and the live cells were immunolabeled with the anti-DPP4 antibody, followed by FACS analysis to measure the cell surface ACE2 or DPP4 levels. (**C**) Huh7 cells were treated with berbamine (10 μM) for 24 h, and cells were then stained with Wheat germ agglutinin (WGA) fluorescein conjugate, followed by flow cytometry analysis. (**D**) The plasma membrane intactness of Huh7 and Vero-E6 after berbamine treatment were monitored by measuring the permeability to trypan blue. (**E**) Huh7 and Vero-E6 cells were treated with berbamine at the indicated concentration for 24 h, followed by PI staining to measure the CC_50_ of berbamine. The graphs represent data from at least three independent experiments. (**F**) EVs were collected from the culture medium of control or berbamine (10 μM)-treated Huh7 cells. The levels of ACE2, DPP4, TSG101, and CD63 in these EVs were determined by immunoblot analysis. (**G**) Cells were treated with berbamine (10 μM) at the indicated concentration for 6 h, after which the cell lysates were subjected to ACE2 or DPP4 immunoblot analysis. (**H**) In TRPML1^L15L/AA-L577L/AA^-expressing HEK293T cells, GFP-TRPML1 was re-routed to the plasma membrane (e. Berbamine significantly inhibited the ML-SA1-induced Ca^2+^ influx in TRPML1^L15L/AA-L577L/AA^-expressing HEK293T cells (right panel). The data were expressed as mean±S.E., n = 90–99 cells. (**I**) Knockdown efficiency of siTRPML1-3 in Hue7 cells was confirmed by qPCR or western blot analysis. (**J**) TRPMLs knockdown significantly inhibited the cell surface DPP4 levels in Huh7 cells as determined by FACS analysis. The graphs represent data from at least three independent experiments. The difference between two groups was analyzed using two-tailed Student's t-test, P<0.05 was considered statistically significant.

Table S1.

**Table S1. Antibodies and Reagents for Flow cytometry, IF and WB**

| **Antibody or Reagent** | **Vendor** | **Catolog No.** | **Species** | **Application** | **Dilution** |
| --- | --- | --- | --- | --- | --- |
| ACE2 | Abcam | ab15348 | Rabbit | Flow cyt, IF | 1:200 |
| DPP4 | Abcam | ab119346 | Mouse | Flow cyt, IF | 1:50 |
| LAMP1 | Cell Signaling | 9091 | Rabbit | IF | 1:200 |
| SARS-CoV-2 NP |  |  | Rabbit | IF | 1:2000 |
| Wheat Germ Agglutinin-488 | Invitrogen | W6748 |  | IF | 1:500 |
| Hochest | Invitrogen | H3569 |  | IF | 1:1000 |
| Propidium Iodide | Invitrogen | P3566 |  | IF | 1:1000 |
| DAPI | Invitrogen | 62247 |  | IF | 1μg/mL |
| HSP 70 | Santa Cruz | sc-24 | Mouse | WB | 1:500 |
| TSG101 | Proteintech | 14497-1-AP | Rabbit | WB | 1:500 |
| CD63 | Proteintech | 25682-1-AP | Rabbit | WB | 1:500 |
| Alix | Proteintech | 12422-1-AP | Rabbit | WB | 1:500 |
| ACE2 | Proteintech | 21115-1-AP | Rabbit | WB | 1:500 |

Table S2.

**Table S2. Sequence of SiRNA**

| **Targets** | **Sequence** |
| --- | --- |
| SiNC | GCAAGAGUAAUGACGAAAU |
| SiTRPML1-1 | CCUUCGCCGUCGUCUCAAA |
| SiTRPML1-2 | AUCCGAUGGUGGUUACUGA |
| SiTRPML1-2 | GAUCACGUUUGACAACAAA |
| SiTRPML2-1 | CCGUGGAAACUGGGUUUGCAGAUUU |
| SiTRPML2-2 | CAACGACGUUGAGCUCGAUUGUGUU |
| SiTRPML3-1 | CAGCGCUGCCCAAUGUCAUCAGGUU |
| SiTRPML3-2 | CCCAGAGACUGAACUUCGUACAUUU |
